# A CRF-based system for recognizing chemical entity mentions (CEMs) in biomedical literature

**DOI:** 10.1186/1758-2946-7-S1-S11

**Published:** 2015-01-19

**Authors:** Shuo Xu, Xin An, Lijun Zhu, Yunliang Zhang, Haodong Zhang

**Affiliations:** 1 Information Technology Supporting Center, Institute of Scientific and Technical Information of China, No. 15 Fuxing Rd., Haidian District, 100038 Beijing, PR China; 2 School of Economics and Management, Beijing Forestry University, No. 35 Qinghua East Rd., Haidian District, 100083 Beijing, PR China; 3 Network Center, Science and Technology Daily, No. 15 Fuxing Rd., Haidian District, 100038 Beijing, PR China

**Keywords:** Chemical Compound and Drug Name Recognition, Conditional Random Fields, Brown Clustering, Natural Language Processing, Word Representations

## Abstract

**Background:**

In order to improve information access on chemical compounds and drugs (chemical entities) described in text repositories, it is very crucial to be able to identify chemical entity mentions (CEMs) automatically within text. The CHEMDNER challenge in BioCreative IV was specially designed to promote the implementation of corresponding systems that are able to detect mentions of chemical compounds and drugs, which has two subtasks: CDI (Chemical Document Indexing) and CEM.

**Results:**

Our system processing pipeline consists of three major components: pre-processing (sentence detection, tokenization), recognition (CRF-based approach), and post-processing (rule-based approach and format conversion). In our post-challenge system, the cost parameter in CRF model was optimized by 10-fold cross validation with grid search, and word representations feature induced by Brown clustering method was introduced. For the CEM subtask, our official runs were ranked in top position by obtaining maximum 88.79% precision, 69.08% recall and 77.70% balanced F-measure, which were improved further to 88.43% precision, 76.48% recall and 82.02% balanced F-measure in our post-challenge system.

**Conclusions:**

In our system, instead of extracting a CEM as a whole, we regarded it as a sequence labeling problem. Though our current system has much room for improvement, our system is valuable in showing that the performance in term of balanced F-measure can be improved largely by utilizing large amounts of relatively inexpensive un-annotated PubMed abstracts and optimizing the cost parameter in CRF model. From our practice and lessons, if one directly utilizes some open-source natural language processing (NLP) toolkits, such as OpenNLP, Standford CoreNLP, false positive (FP) rate may be very high. It is better to develop some additional rules to minimize the FP rate if one does not want to re-train the related models. Our CEM recognition system is available at: http://www.SciTeMiner.org/XuShuo/Demo/CEM.

## Background

There is an increasing interest to improve information access on chemical compounds and drugs (chemical entities) described in text repositories, including scientific articles, patents, health agency reports, or the Web [1]. In order to achieve this goal, it is very crucial to be able to identify chemical entity mentions (CEMs) automatically within text. The recognition of chemical entities is also crucial for other subsequent text processing tasks, such as detection of drug-protein interactions [2], adverse effects of chemical compounds and their associations to toxicological endpoints, or the extraction of pathway and metabolic reaction relations and so on. Though many methods and strategies to recognize chemicals in text have been proposed [3], only a very limited number of publicly accessible CEM recognition systems have been released [4].

The BioCreative (Critical Assessment of Information Extraction Systems in Biology) challenge is a community-wide effort to build an evaluation framework for assessing text mining systems in biological domains [5]. The chemical compound and drug named entity recognition (CHEMDNER) challenge in BioCreative IV was specially designed to promote the implementation of systems that are able to detect mentions of chemical compounds and drugs, which has two subtasks, CDI (Chemical Document Indexing) subtask and CEM (Chemical Entity Mention) subtask. CDI subtask is the task to return a ranked list of chemical entities described within a given documents. CEM subtask is the task to provide for a given document the start and end indices corresponding to all the chemical entities mentioned in the document.

Here, we present the method, the results and recognition system from our participation in the CEM subtask of CHEMDNER challenge [1,6] with some postchallenge systems improvement. In our recognition system, instead of extracting a CEM such as "(+)-antiBP-7,8-diol-9,10-epoxide" as a whole, we regard it as a sequence labeling problem. Our main focus on this improved system was to explore the effectiveness of cost parameter optimization [7,8] and word representation-s [9-11] feature for our approach to CEM subtask. The proposed method combines natural language processing (NLP) strategies with machine learning (ML) techniques to utilize word representations feature from large amounts of relatively inexpensive un-annotated PubMed abstracts along with small amounts of annotated ones.

**Figure 1:**
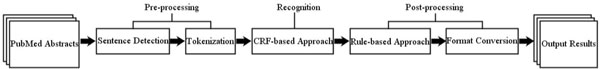
**The system processing pipeline**. The system processing pipeline that includes three major components: pre-processing (sentence detection, tokenization), recognition (CRF-based approach) and post-processing (rule-based approach and format conversion).

As shown in Figure 1, our system first detects sentence boundaries on the PubMed abstracts, and then tokenizes each detected sentence as pre-processing. Next, our system extracts CEMs from text with a conditional random field (CRF) approach [12], followed by some post-processing steps including a rule-based approach and a format conversion step. We describe each step in detail in the following sections. Although current approach has much room for improvement, it produced the top-ranked performance among all submitted runs in the CEM subtask of BioCreative IV CHEMDNER challenge.

The organization of the rest of the article is as follows. In the next section, we describe the results of our submission and post-challenge runs on the CEM subtask of BioCreative IV CHEMDNER challenge. This is followed by discussion and conclusions drawn from our experience. Lastly, our methods employed are explained in detail.

## Results and discussion

We analyzed the training, development and testing data sets and found that there are many nested CEMs in the development set, such as "polysorbate 80" (offset: 1138 to 1152) and "polysorbate" (offset: 1138 to 1149) in the abstract of PMID: 23064325. See Table 1 for more examples of nested CEM pairs. Since linear CRF model, utilized in this article, cannot identify the nested CEMs, we just omit the less spanned CEMs. In addition, there may be some annotation errors in the development set, such as examples in Table 2. We also manually corrected these errors before training our CRF model. Table 3 shows a brief overview of the corrected CHEMDNER corpus. Please see [13] for more details of CEMs annotating, classifying and splitting into training, development and test data sets.

**Table 1:** Nested CEM pairs in the development set of the CHEMDNER corpus.

			Offset		Offset	
				
ID	PMID	T/A	Start	End	Start	End
1	23064325	A	1138	1152	1138	1149

2	23353756	A	12	65	29	65

3	23425199	T	50	66	61	66
		A	56	72	67	72

4	23298577	A	365	381	378	381

5	23368735	A	83	103	97	103
		A	108	119	118	119

6	23562534	A	944	950	944	946

7	23288867	A	1625	1641	1625	1632

8	23500769	A	410	418	410	414

9	23435367	A	118	133	118	125

10	22401710	A	688	696	688	691

11	23350627	A	96	111	101	111
		A	117	130	122	130
		A	467	507	473	475
		A	467	507	482	483

12	23453838	A	467	507	504	507
		A	632	646	640	641
		A	767	782	773	774
		A	843	847	845	847

13	23401298	A	438	502	438	501

14	23567043	A	436	450	444	450

15	23425199	T	50	66	50	60
		A	56	72	56	66

16	22313530	A	306	364	307	364

17	23368735	A	83	103	87	93
		A	108	119	112	114

18	23229510	A	645	739	646	738

19	23562534	A	944	950	947	950

20	23294378	A	584	604	585	603

21	23295645	T	0	33	10	33

22	23435367	A	963	978	963	970

23	23350627	A	96	111	96	100
		A	117	130	117	121

24	23453838	A	467	507	469	471
		A	467	507	479	480
		A	467	507	495	502
		A	632	646	634	636
		A	767	782	769	771
		A	767	782	779	782
		A	909	913	911	913

For each row, the CEM with offset in column 6-7 is nested in the CEM with offset in column 4-5. The CEMs with respective offsets in column 6-7 are omitted directly when training our CRF models.

**Table 2:** Nested CEM pairs in the development set of the CHEMDNER corpus.

			Offset		Offset	
				
ID	PMID	T/A	Start	End	Start	End
1	23412114	A	977	984	977	985

2	23572392	T	42	55	42	56

3	23414800	T	69	89	68	89

4	23411224	A	278	288	277	288

5	23401298	A	438	502	438	501

The offsets in column 4-5 are corrected to the ones in column 6-7.

**Table 3:** The overview of the corrected CHEMDNER corpus in terms of the number of PubMed abstracts (#Articles), the number of CEMs (#CEMs), and the number of CEMs for each of the CEM classes in C = {SYSTEMATIC, IDENTIFIER, FORMULA, TRIVIAL, ABBREVIATION, FAMILY, MULTIPLE, NO CLASS} × means the resulting figure is unknown.

	Training	Development	Test	Background
#Articles	3,500	3,500	3,000	17,000

#CEMs	29,478	29,485	25,351	×

ABBREVIATION	4,538	4,517	4,059	×

FAMILY	4,090	4,212	3,622	×

FORMULA	4,448	4,117	3,443	×

IDENTIFIER	672	639	513	×

MULTIPLE	202	187	199	×

SYSTEMATIC	6,656	6,814	5,666	×

TRIVIAL	8,832	8,967	7,808	×

NO CLASS	40	32	41	×

To evaluate the performance of submitted results, the BioCreative IV competition relied on three performance measures at entity level: recall, precision and F-measure. The recall is the proportion of correct prediction of positive CEMs. The precision is the proportion of predicted CEMs that are actually true CEMs. The F-measure provides a more balanced evaluation by averaging precision and recall. The recall, precision and F-measure are defined formally as follows.

(1) $$r=\frac{TP}{TP+FN}$$

(2) $$p=\frac{TP}{TP+FP}$$

(3) $$F_{\beta }=\left(1+\beta^{2}\right)\frac{p\times r}{\beta^{2}p+r}$$

where *TP *(true positive) is the number of the correct positive predictions, *FN *(false negative) is the number of incorrect negative predictions (type II errors), and *FP *is the number of incorrect positive predictions (type I errors). The balanced F-measure (*β *= 1), the main evaluation metric used for the CEM subtask of the BioCreative IV CHEMDNER competition, can be simplified to:

(4) $$F_{1}=2\frac{p\times r}{p+r}$$

In order to make the best of annotated corpus, we pooled the training and development data sets. The participating teams are allowed to have 5 days to generate up to five different annotations ("runs") for the test set and to submit the annotations to the organizers. Thus, participating teams can utilize different settings, models or methods when gold test annotation set is unknown. We submitted five runs for the CEM subtask, each using the same pipeline, but with different values for the cost parameter in the CRF model [12,14]. Due to time constraints, we just set the cost parameter to each element in {2^−2^, 2^−1^, 2^0^, 2, 2^2^}. Table 4 presents the official performance scores of our submitted runs. Run 5 performed the best in terms of recall and balanced F-measure. Run 1 performed the best in term of precision.

**Table 4:** Official scores for the CEM subtask in the BioCreative IV CHEMDNER competition.

	Run 1	Run 2	Run 3	Run 4	Run 5
cost	2^−2^	2^−1^	2^0^	2	2-

TP	15,821	16,531	16,991	17,328	17,512
FP	1,834	2,007	2,009	2,129	2,211
FN	9,530	8,820	8,360	8,023	7,839

Precision(%)	89.61	89.17	89.43	89.06	88.79
Recall(%)	62.41	65.21	67.02	68.35	69.08
F_1 _score(%)	73.58	75.33	76.62	77.34	77.70

ABBREVIATION	53.90	55.75	56.74	57.63	58.24
FAMILY	59.50	62.20	64.80	66.23	67.28
FORMULA	72.76	74.30	74.88	75.46	75.84
IDENTIFIER	68.62	70.18	69.98	69.79	69.59
MULTIPLE	27.64	32.16	28.64	31.66	31.66
SYSTEMATIC	69.57	72.61	74.32	75.61	76.35
TRIVIAL	58.95	62.73	65.48	67.41	68.26
NO CLASS	51.22	51.22	56.10	58.54	58.54

In fact, the cost parameter trades the balance between over-fitting and under-fitting [12,14]. With larger cost parameter value, CRF tends to over-fit to the given training corpus. From Table 4, one can easily see that the predicted results were significantly influenced by this parameter. In our post-challenge improved systems, 10-fold cross validation at document level is utilized to optimize the cost parameter with grid search [7,8]. Specifically, the pooled training and development data sets are randomly divided into 10 sub-corpus of nearly equal size. For each cost ∈ {2^−3^, 2^−2^, 2^−1^, 2^0^, 2, 2^2^, 2^3^}, a CRF model is induced 10 times, each time leaving out one of the sub-corpuses that is then used to calculate the balanced F-measure. An optimal value of costs is selected from this grid search.

In our post-challenge improved system, we reobtained five runs for the CEM subtask, each using the same pipeline as official submissions, but with different features sets (Table 5). From Table 3, CHEMDNER corpus includes large amounts of relatively inexpensive un-annotated PubMed abstracts. In order to reduce data sparsity and improve further the performance of our system, word representations feature is used in our post-challenge system, since it is a simple and general method for semi-supervised learning [11]. Previous studies [11,15,16] show that word representations feature is a very important feature to improve the balanced F-measure of pre-defined categories of proper names and bio-entity recognition.

**Table 5:** Feature combinations used for post-challenge runs on the CEM subtask.

					Word Representation			
	General Linguistic	Character	Case Pattern	Contextual	500	1000	1500	2000
Run1	√	√	√	√				

Run2	√	√	√	√	√			

Run3	√	√	√	√		√		

Run4	√	√	√	√			√	

Run5	√	√	√	√				√

**Figure 2:**
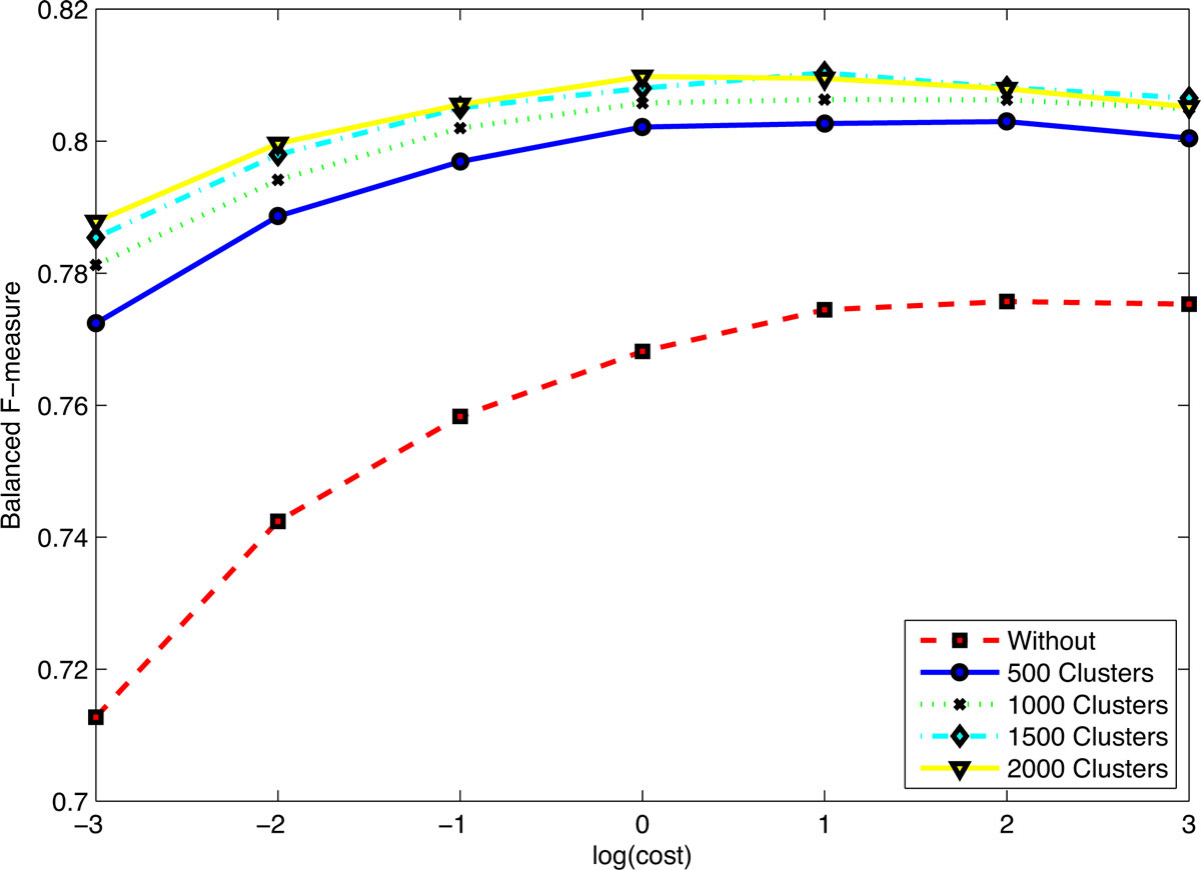
**The balanced F-measure for post-challenge runs with 10-fold cross validation by grid search**.

Here, the training, development, test and background data sets are pooled to induce word representations of each token by Brown clustering method [10,17] with 500, 1000, 1500 and 2000 clusters, respectively. Figure 2 shows the balanced F-measure for postchallenge runs with 10-fold cross validation by grid search [7,8]. Table 6 reports the performance results with the optimal value for the cost parameter. From Figure 2 and by comparing Table 4 and Table 6, it is not difficult to see that the word representations feature improved largely the performance of our system in terms of balanced F-measure and recall, but with a little performance degradation in term of precision. Run 1, Run 4 and Run3 performed the best in term of precision, recall, balanced F-measure, respectively.

**Table 6:** Performance results in our post-challenge improved system for the CEM subtask in the BioCreative IV CHEMDNER competition.

	Run 1	Run 2	Run 3	Run 4	Run 5
**cost**	**2^2^**	**2^2^**	**2**	**2**	**2^0^**

TP	18,025	19,259	19,389	19,495	19,355
FP	2,312	2,671	2,537	2,694	2,505
FN	7,326	6,092	5,962	5,856	5,996

Precision(%)	88.63	87.82	88.43	87.86	88.54
Recall(%)	71.10	75.97	76.48	76.90	76.35
F_1 _score(%)	78.91	81.47	82.02	82.02	81.99

ABBREVIATION	59.77	63.37	64.28	65.85	65.98
FAMILY	72.36	73.55	72.92	73.94	72.97
FORMULA	73.02	73.45	74.12	74.01	74.30
IDENTIFIER	64.72	63.35	66.67	64.33	66.86
MULTIPLE	23.62	32.16	35.18	33.17	30.65
SYSTEMATIC	79.19	82.86	83.25	83.22	82.56
TRIVIAL	71.41	81.76	82.38	82.70	81.56
NO CLASS	53.66	63.41	63.41	68.29	63.41

Though the annotated CEMs are classified into eight classes ℂ = { SYSTEMATIC, IDENTIFIER, FORMULA, TRIVIAL, ABBREVIATION, FAMILY, MULTIPLE, NO CLASS }, the annotations of the individual CEM classes are disregarded in our post-challenge system. In order to highlight the existing gaps in the CEM recognition system, performance results for each category in C are also given in Table 4 and Table 6 in term of precision. As for official performance scores in Table 4, our system worked best on recognizing the FORMULA CEMs for Run 1, Run 2 and Run3, and SYSTEMATIC CEMs for Run 4 and Run 5. From Table 6, one can see that our postchallenge improved system identified SYSTEMATIC CEMs at the best. What's more, it seems be very difficult to recognize MULTIPLE CEMs in both systems. Main reason may be that the number of annotated CEMs is not suffice for the MULTIPLE category (202, 187, 199 for training, development and test data sets, respectively in Table 3).

## Conclusions

In the article, we present our post-challenge system and its performance for the CEM subtask of BioCreative IV CHEMDNER challenge. Our system processing pipeline consists of three major components: preprocessing (sentence detection, tokenization), recognition (CRF-based approach), and post-processing (rulebased approach and format conversion). Our main focus on this improved system was to explore the effectiveness of the cost parameter optimization and word representations feature for the CEM subtask.

In our post-challenge improved system, instead of extracting a CEM as a whole, we regarded it as a sequence labeling problem. The famous CRF model is utilized to solve the sequence labeling problem, whose cost parameter is optimized by 10-fold cross validation with grid search. Different feature types, including general linguistic, character, case pattern, contextual, and word representations features, were exploited for our runs. In order to reduce data sparsity in the annotated training and development data sets, word representations were induced from pooled training, development, test and background data sets by Brown clustering method.

Finkel & Manning [18] proposed a model specifically for recognizing nested named entities by using a discriminative constituency parser. The model explicitly represents the nested structure, allowing entities to be influenced not just by the labels of the tokens surrounding them, as in a CRF, but also by the entities contained in them, and in which they are contained. In ongoing work, the model will be introduced for recognizing nested CEMs.

Though our current system has much room for improvement, our system is valuable in showing that the performance in term of balanced F-measure can be improved largely by utilizing large amounts of relatively inexpensive un-annotated PubMed abstracts. From our practice and lesson, if we directly use some open-source NLP toolkits, such as OpenNLP, Stanford CoreNLP, false positive rate may be very high. It is better to develop some additional rules to minimize the false positive rate if one don't want to re-train the related models.

## Methods

### Pre-processing: sentence detection & tokenization

A sentence detector can identify if a punctuation character marks the end of a sentence or not. Here, the sentence detector in OpenNLP [19] is utilized. However, sentence boundary identification is challenging because punctuation marks are often ambiguous [20]. In order to improve further the performance of the sentence detection, we collected many abbreviations, such as *var*., *sp*., *cv*., *syn*., etc. from the training and development sets. Then we generated several rules, such as if current sentence ends with these abbreviations or comma, or next sentence starts with lower-case letter. In this case, the current and next sentences are merged into a new one.

A tokenizer divides each obtained sentence above into tokens, which usually correspond to words, punctuation, numbers, etc. However, to capture individual components within a CEM, similar to Wei et al. [21], we performed tokenization on a finer level. Specifically, special characters in Table 7, numbers, and Greek symbols are divided as separate tokens. An example is shown in Table 8. Plural upper-case abbreviations are also separated into two tokens, such as "NPs" into "NP" and "s". As a matter of fact, before any pre-processing, we also merged some special characters with the same meaning, such as "≥" vs. "≥", "∗" vs. "*", "≃" vs. " ≅", etc.

**Table 7:** Special characters included in our tokenizer.

( ) [ ] { } > < ≥ ≤ , . / \ ' ™ @ · ©® " : = ≠ ≡ ≈ + - ? _ | ↑ ↓ ← → ↗ ↘ ↙ ↖ ↔ ⇌ ± ~ ∗ % ‰ ∘ # & ; ! £ € ¥ $ × ÷ ‡ † ⋯ ∞ ∨ ∧ √ ⊥ ⊤ ∈ ∃ ⊂ ⊃ ∇ ■ ∂ ∫

**Table 8:** An example of CEM component labels in an excerpt "⋯ [C(8)mim][PF(6)] ⋯ " in PMID: 23265515.

token	⋯	[	C	(
label	O	B	I	I

conditional prob.	⋯	0.994456	0.997241	0.999912

token	8	)	mim	]

label	I	I	I	I

conditional prob.	0.999914	0.999853	0.997244	0.996372

token	[	PF	(	6

label	I	I	I	I

conditional prob.	0.996110	0.995940	0.996733	0.996693

token	)	]	⋯	

label	I	E	O	

conditional prob.	0.825782	0.731261	⋯	

### Recognition: CRF-based approach

As mentioned in Background, we see the CEM recognition problem as a sequence labeling one (see Table 8). As a type of discriminative undirected probabilistic model, CRFs [12,14] are often used for labeling or parsing of sequential data, such as natural language text or biological sequences. CRFs [22-24] has been applied successfully to identify various bio-entities, such as gene, protein and so on, and shown a good performance.

Given token sequence $\vec{x}=\left(x_{1},\;x_{2},\cdots ,x_{N}\right)$, CRF defines the conditional probability distribution $\mathrm{Pr}(\vec{y}|\vec{x})$ of label sequence $\vec{y}=\left(y_{1},y_{2},\cdots ,y_{N}\right)$ as follows.

(5) $$\text{Pr}\left(\vec{y}|\vec{x}\right)\;\;\propto \;\;\text{exp}\left({\vec{w}}^{T}\vec{f}\left(y_{n},y_{n-1},\vec{x}\right)\right)$$

Here, $\vec{w}={\left(w_{1},w_{2},\cdots ,w_{M}\right)}^{T}$ is a global feature weight vector, $\vec{f}\left(y_{n},y_{n-1},\vec{x}\right)={\left(f_{1}\left(y_{n},y_{n-1},\vec{x}\right),f_{2}\left(y_{n},y_{n-1},\vec{x}\right),\cdots ,f_{M}\left(y_{n},y_{n-1},\vec{x}\right)\right)}^{T}$ is a local feature vector function, and M is the number of feature functions. The weight vector w can be obtained from the training and development sets by a limited-memory Broyden-Fletcher-Goldfarb-Shanno (L-BFGS) [25] method.

The traditional BIEO label set is used in our post-challenge improved system. That is to say, each token is labeled as being the beginning of (B), the inside of (I), the end of (E) or entirely outside (O) of a span of interest. Here, CRF++ [26] is adopted for the actual implementation. In CRF++, there are 4 major parameters ("-a", "-c", "-f" and "-p") to control the training condition. In our submitted predictions and post-challenge ones, the parameters "-a", "-f" and "- p" were consistently set to CRF-L2, 2 and 4, respectively. The option "-c" is optimized with 10-fold cross validation, as introduced above.

### Features for our CRF model

Our system exploits four different types of features:

#### General linguistic features

Our system includes the original uni-tokens and bi-tokens, as well as stemmed uni-tokens, bi-tokens and tri-tokens, as features using the Porter's stemmer [27] from Stanford CoreNLP [28].

#### Character features

Since many CEMs contain numbers, Greek letters, Roman numbers, amino acids, chemical elements, and special characters, our system calculates several statistics as features for each token, including its number of digitals, number of upper- and lower-case letters, number of all characters and presence or absence of specific characters or Greek letters, Roman numbers, amino acids, or chemical elements.

#### Case pattern features

Similar to [21], any upper case alphabetic character is replaced by 'A', any lower case one is replaced by 'a', and any number (0-9) is replaced by '0'. Moreover, our system also merge consecutive letters and numbers and generated additional single letter 'a' and number '0' features.

#### Contextual features

For each token, our system includes a combination of the current output token and previous output token (bigram).

#### Word representation features

One common approach to inducing unsupervised word representation is to use clustering, perhaps hierarchical, such as Brown clustering method [17], Collobert and Weston embeddings [29], hierarchical log- bilinear model (HLBL) embeddings [30] and so on. Here, the Brown clustering method is used. The implementation of Brown clustering method by Liang [31] is adopted in our post-challenge system.

The result of running the Brown clustering method is a binary tree, where each token occupies a single leaf node, and where each leaf node contains a single token. The root node defines a cluster containing the entire token set. Interior nodes represent intermediate size clusters containing all of the tokens that they dominate. Thus, nodes lower in the binary tree correspond to smaller token clusters, while higher nodes correspond to larger token clusters. According to Huffman coding [32], a particular token can be assigned a binary string by following the traversal path from the root to its leaf, assigning a 0 for each left branch, and a 1 for each right branch.

Intuitively, the Brown clustering method will merge the tokens with similar contexts into the same cluster. Thus, the more similar the prefix of the token's Huffman coding, the more similar the tokens. Table 9 shows some token examples and their binary string representations with 500 clusters. Let's take Table 9 as an example. According to main idea of the Brown clustering method, the token "interpeak" (01100110110) is more similar than the token "aquaporine" (01101110011) with the token "florbetapir" (0110011010).

**Table 9:** Sample tokens and their resulting binary string representations with 500 clusters.

ID	Token	Binary String
1	gracile	010011

2	quintile	010010

3	florbetapir	0110011010

4	interpeak	01100110110

5	aquaporine	01101110011

### Post-processing: rule-based approach & format conversion

On closer examination, we find that the results of CRF approach include some false positive CEMs, such as "25(3), 186-193", "1-D, 2-D" and so on. So, we developed several additional regular expresses to remove them. In addition, our post-processing step also helps adjust text spans of CEMs, such as adding a missing closing parenthesis, such as "[4Fe-4S](2+" into "[4Fe-4S](2+)". All of the adjustment rules are listed in Table 10. Here, #(·, str) means the number of occurrences of the string str in the interested CEM, right(·, *n*) and left(·, *n*) denote the substring with the length of *n *right or left to the interested CEM, and offset(·, start) and offset(·, left) indicate the start or end offset of the interested CEM. Let's take the first row in Table 10 as an example. It means that if the number of the occurrences of "(" is higher than that of ")" in the interested CEM, and if the substring with the length of 1 right to the interested CEM is ")", then start offset of the interested CEM is moved one character further to the right.

**Table 10:** The Adjustment Rules of the Text Spans in the BioCreative IV CHEMDNER competition.

ID	IF Condition	Action
1	#(·, "(") == #(·, ")") + 1 ∧ right(·, 1) == ")"	offset(·, end) = offset(·, end) + 1

2	#(·, "(") == #(·, ")") - 1 ∧ left(·, 1) == "("	offset(·, start) = offset(·, start) - 1

3	#(·, "[") == #(·, "]") + 1 ∧ right(·, 1) == "]"	offset(·, end) = offset(·, end) + 1

4	#(·, "[") == #(·, "]") - 1 ∧ left(·, 1) == "["	offset(·, start) = offset(·, start) - 1

5	#(·, "{") == #(·, "}") + 1 ∧ right(·, 1) == "}"	offset(·, end) = offset(·, end) + 1

6	#(·, "{") == #(·, "}") - 1 ∧ left(·, 1) == "{"	offset(·, start) = offset(·, start) - 1

7	#(·, "<sc>") == #(·, "</sc>") + 1 ∧ right(·, 5) == "</sc>"	offset(·, end) = offset(·, end) + 5

8	#(·, "<sc>") == #(·, "</sc>") - 1 ∧ left(·, 4) == "<sc>"	offset(·, start) = offset(·, start) - 4

9	#(·, "<i>") == #(·, "</i>") + 1 ∧ right(·, 4) == "</i>"	offset(·, end) = offset(·, end) + 4

10	#(·, "<i>") == #(·, "</i>") - 1 ∧ left(·, 3) == "<i>"	offset(·, start) = offset(·, start) - 3

11	#(·, "<sup>") == #(·, "</sup>") + 1 ∧ right(·, 6) == "</sup>"	offset(·, end) = offset(·, end) + 6

12	#(·, "<sup>") == #(·, "</sup>") - 1 ∧ left(·, 5) == "<sup>"	offset(·, start) = offset(·, start) - 5

13	#(·, "<sub>") == #(·, "</sub>") + 1 ∧ right(·, 6) == "</sub>"	offset(·, end) = offset(·, end) + 6

14	#(·, "<sub>") == #(·, "</sub>") - 1 ∧ left(·, 5) == "<sub>"	offset(·, start) = offset(·, start) - 5

#(·, *str*) means the number of occurrences of the string *str *in the interested CEM, right(·, *n*) and left(·, *n*) denote the substring with the length of *n *right or left to the interested CEM, and offset(·, start) and offset(·, left) indicate the start or end offset of the interested CEM.

Finally, we converted the recognized CEMs into the official format with the resulting confidence scores. In our system, the confidence score is simply set to averaged conditional probably of each tokens composed of the interested CEM, formally defined as follows.

(6) $$\text{score}(\text{CEM})=\frac{1}{\left|\text{CEM}\right|}\sum_{t\in \text{CEM}}\text{CondProb}(\text{t})$$

where |CEM| means the number of token components of a CEM. Take "[C(8)mim][PF(6)]" in Table 8 as an example. Its confidence score is calculated as follows.

(7) $$\begin{array}{l} \;\text{score}([\text{C}(8)\text{mim}][\text{PF}(6)])\\ =\frac{1}{13}\sum_{t\in [\text{C}(8)\text{mim}][\text{PF}(6)]}\text{CondProb}(\text{t})\\ =0.963655 \end{array}$$

## Competing interests

The authors declare that they have no competing interests.

## Authors' contributions

SX and YZ developed the CEM recognition system. SX and HZ conducted extensive experiments and drafted the manuscript. LZ and XA conceived of the supporting projects, and participated in the resulting design and coordination and helped draft the manuscript. All authors read and approved the final manuscript.

## Declarations

This work was supported partially by Fundamental Research Funds for the Central Universities: Research on Forest Property Circulation Mechanism in Collective Forest Area (JGTD2014-04), Beijing Forestry University Young Scientist Fund: Research on Econometric Methods of Auction with their Applications in the Circulation of Collective Forest Right (BLX2011028), the National Science Foundation of China: Research on Technology Opportunity Detection based on Paper and Patent Information Resources (71403255), Key Technologies R&D Program of Chinese 12th Five-Year Plan (2011-2015): STKOS Collaborative Construction System and Auxiliary Tool Development (2011BAH10B02), and Key Work Project of Institute of Scientific and Technical Information of China (ISTIC): Intelligent Analysis Service Platform and Application Demonstration for Multi-Source Science and Technology Literature in the Era of Big Data (ZD2014-7-1).

This article has been published as part of *Journal of Cheminformatics *Volume 7 Supplement 1, 2015: Text mining for chemistry and the CHEMDNER track. The full contents of the supplement are available online at http://www.jcheminf.com/supplements/7/S1.
